# Molecular and pharmacological evidence for a facilitatory functional role of pre-synaptic GLUK2/3 kainate receptors on GABA release in rat trigeminal caudal nucleus

**DOI:** 10.1002/j.1532-2149.2012.00122.x

**Published:** 2012-03-06

**Authors:** I Samengo, D Currò, P Navarra, V Barrese, M Taglialatela, M Martire

**Affiliations:** 1 Institute of Pharmacology, Catholic University of Sacred HeartRome, Italy; 2 Division of Pharmacology, University of Naples Federico IINaples, Italy; 3 Department of Health Sciences, University of MoliseCampobasso, Italy

## Abstract

**Background:**

Gamma-aminobutyric acid (GABA) and glutamate (GLU) are involved in nociceptive signals processing in the trigeminal system. In this study, we investigated the influence of excitatory transmission on GABA release in nerve terminals isolated from the rat trigeminal caudal nucleus (TCN).

**Methods:**

We utilize biochemical (superfused synaptosomes loaded with [^3^H]GABA) and morphological (immunofluorescence experiments with specific antibody) techniques.

**Results:**

Our results show that GLU potentiates the release of [^3^H]GABA evoked by 9, 15 and 30 mM [K^+^]_e_; 15 mM [K^+^]_e_-evoked [^3^H]GABA release was also reinforced by domoate and kainate (KA), two naturally occurring GLU-receptor agonists. The enhancement of 15 mM [K^+^]_e_-evoked [^3^H]GABA release produced by 100 μM KA was abolished by NBQX, a mixed AMPA/KA receptor antagonist, but was not affected by GYKI52466, a selective AMPA receptor antagonist. ATPA, a selective agonist for KA receptors containing the GLUK1 subunit, had no effect on depolarization-induced [^3^H]GABA release, and UBP310, which selectively antagonizes these same receptors, failed to reverse the KA-induced potentiation of 15 mM [K^+^]_e_-evoked [^3^H]GABA release. The KA-induced potentiation was also unaffected by concanavalin A (10 μM), a positive allosteric modulator of GLUK1- and GLUK2-containing KA receptors.

Immunofluorescence experiments revealed that GABAergic nerve terminals in the TCN differentially expressed GLUK subunits, with GLUK2/3-positive terminals being twice more abundant than GLUK1-containing synaptosomes.

**Conclusions:**

These findings indicate that pre-synaptic KA receptors facilitating GABA release from TCN nerve terminals mainly express GLUK2/GLUK3 subunits, supporting the notion that different types of KA receptors are involved in the various stages of pain transmission.

## 1. Introduction

Primary afferent neurons that transmit nociceptive information from the orofacial region to the central nervous system (CNS) terminate in the brainstem trigeminal caudal nucleus (TCN) (Hu et al., 1981), which is often referred to as the ‘medullary dorsal horn’ because it is quite similar to the dorsal horn of the spinal cord in terms of morphological organization and connectivity (Bereiter et al., 2000).

**What's already known about this topic?**

- Gamma-aminobutyric acid (GABA) controls neural transmission between first- and second-order neurons in the trigeminal system by reducing the excitability of trigeminal caudal nucleus glutamatergic neurons.

**What does this study add?**

- GABA release is facilitated by pre-synaptic kainate receptor subtypes (GLUK2/GLUK3).
- These are pharmacologically and morphologically different from those (GLUK1/GLUK5) expressed on the somata of second-order neurons.
- These findings suggest that development of selective GLUK2/GLUK3 receptor agonists might open new avenues for treatment of migraines.

At both spinal and supraspinal levels, glutamate (GLU) is a primary sensory neurotransmitter. Although GLU effects in the TCN are primarily mediated by the activation of ionotropic post-synaptic receptors (Storer and Goadsby, 1999), pre-synaptic nerve terminals displaying immunoreactivity for GLU have also been found in lamina II of the TCN (Iliakis et al., 1996), an anatomical region containing a high density of binding sites for *N*-methyl-D-aspartate (NMDA), (±)-α-amino-3-hydroxy-5-methylisoxazole-4-propionic acid hydrobromide (AMPA) and kainate (KA) receptors (Tallaksen-Greene et al., 1992).

A large body of experimental evidence suggests that ionotropic glutamatergic receptors of the KA subtype are involved in both pre- and post-synaptic sensory transmission and in the modulation of pain at the spinal and supraspinal levels (Huettner, 1990; Sahara et al., 1997; Li et al., 1999). The role of KA receptors in nociception and/or chronic pain is supported by the selective transcriptional up-regulation of KA-receptor subunit genes, leading to changes in subunit composition of KA receptors in a rat model of inflammatory hyperalgesia (Guo et al., 2002). KA receptors located on the central endings of dorsal root ganglia neurons regulate glutamatergic transmission (Kerchner et al., 2001a, 2002; Youn and Randic, 2004). KA receptors are also found post-synaptically on dendrites and cell bodies in the superficial laminae of the rat spinal dorsal horn (Li et al., 1999; Hwang et al., 2001; Lucifora et al., 2006). While pre-synaptic KA receptors expressed on spinal cord inhibitory interneurons activate inhibitory transmission in a dose-dependent and biphasic manner, activation of pre-synaptic GABA_B_ autoreceptors exerts inhibitory effects on GABAergic neurotransmission (Kerchner et al., 2001b).

The inhibitory neurotransmitter γ-aminobutyric acid (GABA) is an antinociceptive transmitter that affects various levels of pain transmission within the CNS (Malcangio and Bowery, 1996). Numerous pharmacological agents and physical stimuli produce analgesia by activating GABA_A_ and GABA_B_ receptors, while GABA_A_ antagonists are known to produce allodynia (Enna and McCarson, 2006). In addition, reduced endogenous GABAergic activity and loss of GABAergic neurons and GABA transporters in the spinal cord have been observed in chronic pain states (Munro et al., 2009; Gwak and Hulsebosch, 2011).

In this study, we investigated the modulatory effects of the excitatory glutamatergic neurotransmission on the release of GABA from synaptosomes from the lower brainstem containing the TCN. Our findings indicate that depolarization-induced GABA release in the TCN is facilitated by a pre-synaptic KA receptor containing GLUK2/3 subunits.

These results highlight GLUK2/3 receptors as relevant targets for the enhancement of inhibitory neurotransmission in the TCN, an observation which might prove useful to design novel pharmacological strategies to control pain states originating from the orofacial region.

## 2. Methods

### 2.1 Animals

Adult male Wistar rats (weighing 200–250 g) were used in the experiments. Animals were housed under constant conditions of temperature (22 ± 1 °C) and relative humidity (50%) with a regular light-dark schedule (lights on from 7:00 a.m. to 7:00 p.m.) and free access to food and water. All animal experiments performed in this study were carried out in accordance with the European Community Council Directive of 1986 (86/609/EEC) and were approved by the Ethics Committee of the Catholic University of the Sacred Heart. Special care was taken to minimize suffering and the number of animals used.

### 2.2 Preparation of synaptosomes

Animals were sacrificed, and the brain and upper portion of the spinal cord were rapidly removed and immersed in ice-cold medium (described in detail below). We eliminated the cerebral cortex and cerebellum and sectioned the tissue block containing the brainstem at the level of the obex and 0.4 cm below it (D'Amico et al., 2010). The lateralmost portions (right and left) of the resulting tissue block, which contained the TCN, were then isolated and used to prepare synaptosomes. For release experiments, crude synaptosomes were used and prepared as previously described (Martire et al., 2004). In brief, the brain tissue was placed in 40 volumes of 0.32 M sucrose buffered to a pH of 7.2 with phosphate and homogenized (12 strokes at 900 rpm in ∼1 min) with a glass-Teflon tissue grinder (clearance 0.25 mm). The homogenate was centrifuged at 1000 *g* for 5 min, and the synaptosomes were isolated from the supernatant by centrifugation at 12,000 *g* for 20 min. All of the above procedures were performed at 0–4 °C. The synaptosome pellet was resuspended in a physiological medium (standard medium) containing (in mM) NaCl 125, KCl 3, MgSO_4_ 1.2, CaCl_2_ 1.2, NaH_2_PO4 1.0, NaHCO_3_ 22, and glucose 10 (pH 7.40), and oxygenated with 95% O_2_/5% CO_2_. For immunofluorescence experiments, purified synaptosomes were used and prepared as previously described (Luisi et al., 2009). Briefly, the tissue was homogenized in 10 volumes of 0.32 M sucrose and centrifuged at 1000 *g* for 10 min. The supernatant was gently stratified on a discontinuous Percoll gradient (3%, 10%, 15% and 23% v/v) and centrifuged at 32,000 *g* for 5 min. The layers between 15% and 23% Percoll bands (synaptosomal fraction) were collected and washed twice with a physiological medium (solution A); finally, the synaptosomal pellet was resuspended in phosphate-buffered saline (PBS).

### 2.3 Release experiments

Synaptosomes were incubated for 15 min with 0.03 μM γ-[2,3-^3^H(N)]aminobutyric acid ([^3^H]GABA) in standard medium in an atmosphere of 95% O_2_/5% CO_2_ at 37 °C. Amino-oxyacetic acid (50 μM) was also present in the medium to prevent GABA catabolism. At the end of incubation, identical aliquots of the synaptosome suspension (with protein contents ranging from 0.8 to 1.2 mg depending on the experiment) were distributed as monolayers on microporous filters (0.8 μm Millipore) positioned at the bottom of a set of parallel superfusion chambers, which were maintained at 37 °C (Raiteri and Raiteri, 2000). The suspension was then washed with 3 × 10 mL of standard medium at 37 °C under moderate vacuum filtration and superfused for a total of 49 min with standard medium aerated with 95% O_2_/5% CO_2_ (rate 0.6 mL/min). The first 30 min of perfusion (minutes 1–30) were an equilibration period during a constant rate of basal release was reached. This was followed by an 8-min pre-stimulation phase (minutes 30–38), during which basal release was measured (minutes 35–37). The next 3 min (minutes 38–41) constituted the stimulation phase, during which synaptosomes were exposed to the depolarizing stimulus (i.e. standard medium with equimolar substitution of KCl for NaCl – generally 15 mM), with or without test agonists. Perfusion was then resumed with standard medium alone (without depolarizing stimulus or test substances) (minutes 41–49, post-stimulation phase). Antagonists, when used, were added to the medium 4 min before the agonists and remained there through the end of the stimulation phase (minutes 34–41).

Fractions of the superfusate were collected every 2 min, starting from minute 35 of the pre-stimulation phase, and radioactivity was counted in each fraction and in the superfused synaptosomes themselves. (More detailed descriptions of the experimental methods are provided in the figure legends.)

### 2.4 Immunofluorescence experiments

Synaptosomes were plated on poly-D-lysine-coated coverslip for 1 h at 37 °C in the absence of CO_2_ and then fixed in 4% paraformaldehyde (w/v) pH 7.4 for 15 min, followed by several washes with PBS (pH 7.4). Synaptosomes were then incubated at room temperature for 30 min with the following primary antibodies: (1) mouse anti-vesicular GABA transporter (VGAT; dil. 1:100, Synaptic Systems, Goettingen, Germany); (2) rabbit anti-KA receptor subunits GluK2/3 (dil 1:100, Millipore, Temecula, CA, USA); and (3) goat anti-KA receptor subunit GluK1 (dil 1:100, Santa Cruz Biotechnology, Santa Cruz, CA, USA). Synaptosomes were then washed in PBS and incubated at room temperature for 30 min with a mixture of the following fluorescent secondary antibodies (1:100; Jackson ImmunoResearch Laboratories, Inc., West Grove, PA, USA.), depending on the experimental protocol: (1) anti-goat IgG conjugated to CY3; (2) anti-rabbit IgG conjugated to DyLight 488; and (3) anti-mouse IgG conjugated to DyLight 549 or CY2. Coverslips were then washed in PBS, mounted with Fluoro Care antifade mountant (Biocare Medical, Concord, CA, USA), and analysed using a Zeiss LSM 510 Meta argon/krypton laser scanning confocal microscope. Images were acquired using the multi-track system to avoid crosstalk among channels, with fluorescence excitation lines set at 488 and 543 nm and emission filters BP505-550 (for CY2 and DyLight 488 fluorochromes) and BP560-615 (for CY3 and DyLight 549 fluorochromes). Optical sections were confocally captured using a 63× oil immersion objective (PlanApochromat, NA = 1.4), with a maximal confocal zoom factor of 3, fixed box sizes of 512 × 512 pixels, pinhole below 1 Airy unit. The colour scheme used was green for GluRs- and red for VGAT-labelled structures. Computer-assisted quantitative evaluation of signal overlap by different secondary antibodies in distinct synaptosomal particles was taken as a measure of co-localization, using the Image-J software (http://rsb.info.nih.gov/ij/), as previously described (Martire et al., 2010). In particular, pre-synaptic nerve endings were identified by size (0.5–2 μm); in these identified particles (each spanning about 20 × 20 pixels), a threshold value of fluorescence intensity was applied to allow classification as positive or negative for staining with each of the antibodies; co-localization was defined as positive staining of both markers in a single particle. This procedure was chosen to minimize possible differences in the sub-cellular localization among the different markers.

### 2.5 Calculations

The amount of radioactivity found in a given superfusate fraction was expressed as a percentage of the total tritium present in the synaptosomes at the beginning of the respective collection period. The percentage of radioactivity found in each fraction was plotted against perfusion time (min) to evaluate the time course of neurotransmitter release under different conditions. To evaluate the effects of test drugs, we compared the integrated areas under the time/release curves (AUCs) obtained in the presence of the test drug and under control conditions (assessed in parallel). The results were expressed as an AUC value or as percentage increases or decreases relative to control values. Reported data represent means ± standard deviation (SD) of the given number of experiments (*n*). Data were analysed with one-way analysis of variance followed by Dunnett's multiple comparison test. *p*-values < 0.05 were accepted as significant.

In the analysis of immunofluorescence experiments, data are expressed as mean ± SD of the results obtained from three to six different coverslips each recorded in a different experimental session. For each coverslip, between 10 and 45 individual synaptosomes were monitored. Each experimental session was performed on one synaptosomal preparation derived from two animals.

### 2.6 Drugs

[^3^H]GABA (specific activity 35 Ci/mmol) was purchased from Perkin Elmer Life and Analytical Sciences (Boston, MA, USA). AMPA; 2-carboxy-3-carboxymethyl-4-isopropenylpyrrolidine (kainic acid monohydrate); domoic acid [domoate (DO)]; glutamic acid (GLU); NMDA; 2,3-dioxo-6-nitro-1,2,3,4-tetrahydrobenzo[f]quinoxaline −7-sulfonamide disodium salt (NBQX); 4-(8-methyl-9H-1,3-dioxolo[4,5-h][2,3]benzodiazepin-5-yl)-benzenamine hydrochloride (GYKI52466); concanavalin A (Con A); and (*RS*)-2-amino-3-(3-hydroxy-5-*tert*-butylisoxazol-4-yl) propionic acid (ATPA) were purchased from Sigma-Aldrich (St. Louis, MO, USA). (*S*)-1-(2-amino-2-carboxyethyl)-3-(2-carboxythiophene-3-yl-methyl)-5-methylpyrimidine-2,4-dione (UBP310) was obtained from Tocris Cookson (Bristol, UK).

## 3. Results

**Figure 1:**
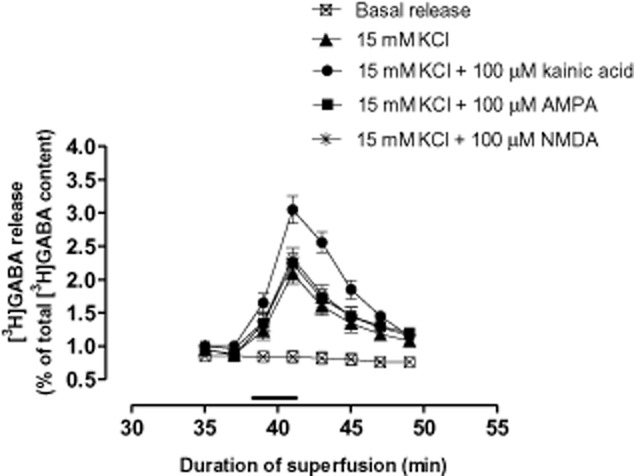
Effects of ionotropic glutamate receptor agonists [kainate (KA), AMPA and *N*-methyl-D-aspartate (NMDA)] on 15 mM [K^+^]_e_-evoked release of [^3^H]GABA from rat superfused caudal brainstem synaptosomes. Synaptosomes (pre-labelled with [^3^H]GABA and superfused with standard medium at a flow rate of 0.6 mL/min) were depolarized with a 180-s pulse of 15 mM [K^+^]_e_ at superfusion minute 38. Agonists were added with the depolarizing stimuli. Superfusate fractions were collected every 2 min, starting from superfusion minute 35 (pre-stimulation). Radioactivity was counted in each fraction and in the superfused synaptosomes themselves to estimate [^3^H]GABA release, which was expressed as a percentage of the total tritium content at the outset of the collection period. Each point represents the mean ± standard deviation of three to four different experiments run in triplicate. Error bars are absent when the error was smaller than the symbol used in the graph. To assess the effects of KA, AMPA and NMDA, the area under the time/release curve (AUC) recorded in the presence of the agonist was compared with the mean AUC recorded in controls (exposed to 15 mM [K^+^]_e_ in the absence of agonists) (Dunnett's test after significant analysis of variance.

**Figure 2:**
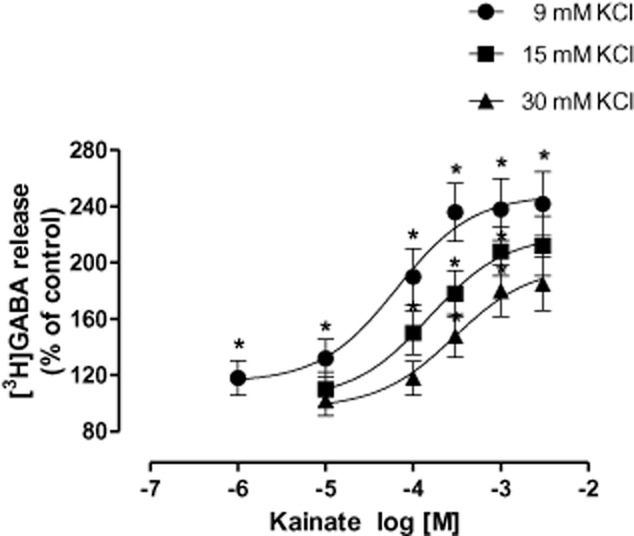
Concentration-dependent effects of kainate (KA) on [^3^H]GABA release evoked by different depolarizing stimuli. KA was added together with the depolarizing stimulus (9, 15 and 30 mM [K^+^]_e_), which remained in the medium for 3 min (superfusion minutes 38–41). Results are expressed as percentage increases in the [K^+^]_e_-evoked release of [^3^H]GABA. Each point represents the mean ± standard deviation of three to five different experiments run in triplicate. *p < 0.01 versus [K^+^]_e_ -evoked efflux in the absence of KA.

To determine whether GABAergic terminals in the TCN express functional ionotropic GLU receptors, we superfused [^3^H]GABA-pre-labelled rat caudal brainstem synaptosomes with KA, AMPA or NMDA (100 μM each) under basal conditions or in the presence of 15 mM [K^+^]_e_. KA, AMPA or NMDA (100 μM each) did not affect the spontaneous (basal) release of [^3^H]GABA (data not shown); by contrast, as shown in Fig. 1, KA, but not AMPA or NMDA, was found to potentiate 15 mM [K^+^]_e_-evoked [^3^H]GABA release (EC_50_ = 150 ± 12.98 μM; Emax = 220 ± 23.87%) (Fig. 2).

The magnitude of the KA-induced potentiation of [K^+^]_e_-evoked [^3^H]GABA release was related to the degree of depolarization (Fig. 2). When synaptosomes were depolarized with a lower (9 mM) [K^+^]_e_ concentration, the concentration–response curve for KA was shifted to the left (EC_50_ = 65 ± 6.95 μM; Emax = 248.50 ± 21.45%); by contrast, when synaptosomes were depolarized with 30 mM [K^+^]_e_, KA was less potent and effective (EC_50_ = 290 ± 28.55 μM; Emax = 198 ± 20.42%). In subsequent experiments, we decided to use a [K^+^]_e_ concentration of 15 mM [K^+^]_e,_ since: (1) [^3^H]GABA released from superfused rat synaptosomes depolarized with relatively low (9–15 mM) [K^+^]_e_ concentrations is entirely dependent on external Ca^2+^; (2) 15 mM [K^+^]_e_ is the concentration most frequently used in experimental studies of this type and it is considered to be a physiological stimulus (Raiteri et al., 2002); and (3) 15 mM [K^+^]_e_-evoked responses were larger and more reproducible than those triggered by 9 mM [K^+^]_e_, therefore allowing a more precise quantification of the observed pharmacological effects.

**Figure 3:**
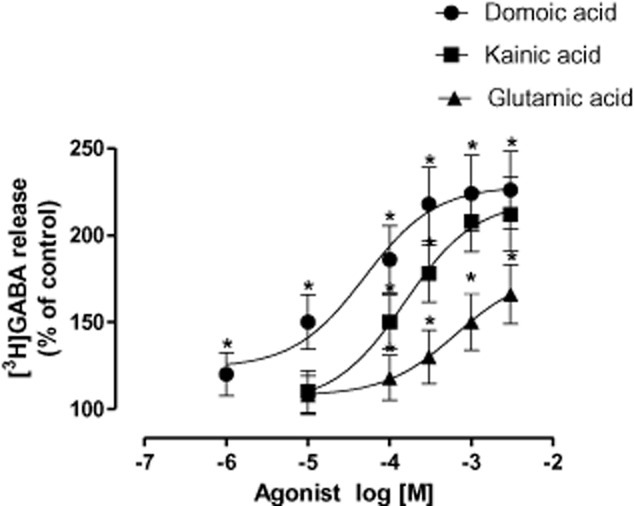
Concentration dependency of the potentiating effects of kainate (KA) (0.010–3 mM), domoate (DO) (0.0010–3 mM) and glutamate (GLU) (0.010–3 mM) on 15 mM [K^+^]_e_-evoked [^3^H]GABA release. The ionotropic GLU-receptor agonists were applied with the depolarizing stimulus (15 mM [K^+^]_e_) at the beginning of superfusion minute 38 and left in the medium for 3 min (through minute 41). Results are expressed as percentage increases in the [K^+^]_e_-evoked release of [^3^H]GABA [ratio of the area under the time–release curve (AUC) observed in the presence of [K^+^]_e_ + KA, [K^+^]_e_ + DO or [K^+^]_e_ + GLU to the AUC in the presence of [K^+^]_e_ alone]. Each point represents the mean ± standard deviation of three to four different experiments run in triplicate. Each point is derived from an AUC found to be significantly different from that of controls (15 mM [K^+^]_e_ without agonists). Therefore, analysis of variance was performed for each agonist compared with its respective control group. *p < 0.01 versus control [K^+^]_e_-evoked efflux.

KA (0.010-3 mM), GLU (0.010–3 mM) and the KA-receptor agonist DO (0.0010–3 mM) increased 15 mM [K^+^]_e_-evoked [^3^H]GABA release in a concentration-dependent manner (Fig. 3). When compared with KA, DO exhibited similar maximal efficacy (Emax = 228.40 ± 19.90%), but higher potency (EC_50_ = 49 ± 4.15 μM). At the maximal concentration tested (3 mM), GLU potentiated evoked release by 166 ± 16.60%. The potency rank order was therefore DO > KA > GLU. Similar to KA, GLU and DO did not affect the spontaneous efflux of [^3^H]GABA (data not shown).

**Figure 4:**
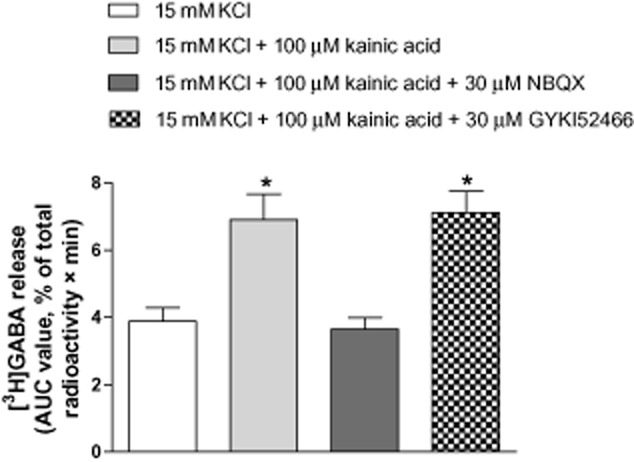
Effects of NBQX, a non-selective AMPA/kainate (KA) receptor antagonist, and GYKI52466, a selective non-competitive antagonist of AMPA receptors, on KA-induced potentiation of 15 mM [K^+^]_e_-evoked [^3^H]GABA release from rat caudal brainstem synaptosomes. NBQX (30 μM) or GYKI52466 (30 μM) was present in the medium before (minutes 34–38) and during synaptosome exposure to KA (100 μM) and 15 mM [K^+^] (3 min, from minute 38 to 41). Results are reported as time–release areas under the curve (AUCs) (after subtraction of the AUC representing basal release) obtained under the experimental different conditions. Each point represents the mean ± standard deviation of three different experiments run in triplicate. *p < 0.01 versus 15 mM [K^+^]_e_-evoked efflux in the absence of KA and antagonists.

To verify that facilitation of the 15 mM [K^+^]_e_-evoked release of [^3^H]GABA induced by KA was indeed mediated by KA receptors, we evaluated the KA-induced potentiation in the presence of NBQX, a non-selective AMPA/KA-receptor antagonist, and GYKI52466, a selective non-competitive antagonist of AMPA receptors. The facilitatory action of KA (100 μM) on 15 mM [K^+^]_e_-evoked [^3^H]GABA release was completely reversed by 30 μM NBQX, but it was unaffected by 30 μM GYKI52466 (Fig. 4). Neither of the antagonists tested (each at a concentration of 30 μM) affected basal [^3^H]GABA release (data not shown).

**Figure 5:**
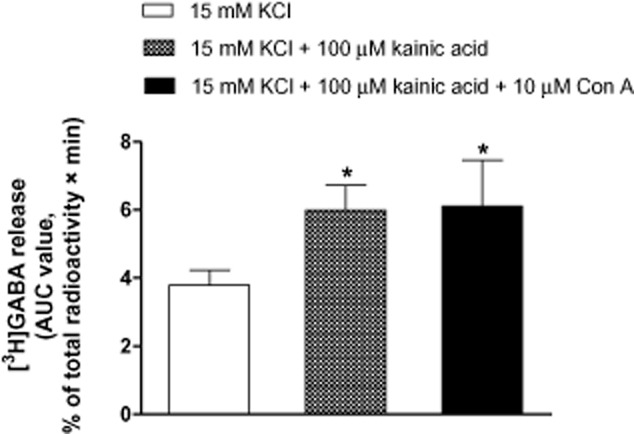
Lack of effect of concanavalin A (Con A), an allosteric activator of kainate (KA) receptors, on KA-induced potentiation of 15 mM [K^+^]_e_-evoked [^3^H]GABA release from rat caudal brainstem synaptosomes. Con A (10 μM) was added together with KA (100 μM) and the depolarizing stimulus at the beginning of superfusion minute 38. The data presented are means ± standard deviation of three experiments run in triplicate. *p < 0.01 versus 15 mM [K^+^]_e_-evoked efflux in the absence of KA and Con A.

KA receptors can also be characterized by their responses to drugs that modify their desensitization process, such as the plant lectin Con A, which is an allosteric activator of GLUK1/2-containing KA receptors. Con A (10 μM) failed to affect both basal (data not shown) and 15 mM [K^+^]_e_-evoked [^3^H]GABA release induced by KA (Fig. 5).

**Figure 6:**
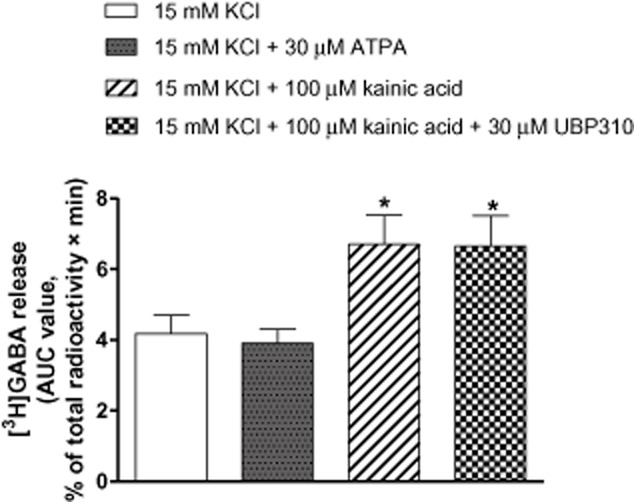
Lack of effect of ATPA, a potent selective agonist for GLUK1-containing kainate (KA) receptors, on 15 mM [K^+^]_e_-evoked [^3^H]GABA release from rat caudal brainstem synaptosomes and of UBP310, a potent GLUK1 receptor antagonist, on KA potentiation of this release. KA and ATPA were added to the medium with the depolarizing stimulus; UBP310 was present in the medium 4 min before and during exposure to KA and [K^+^]_e_. The data presented are means ± standard deviation of three experiments run in triplicate. *p < 0.01 versus 15 mM [K^+^]_e_-evoked efflux in the absence of KA, ATPA and UBP310. AUC, area under the time/release curve.

In order to identify the KA-receptor subtype responsible for the KA-induced potentiation of 15 mM [K^+^]_e_-evoked [^3^H]GABA release, we tested the effect of ATPA, a potent, selective agonist for GLUK1-containing KA receptors, and of UBP310, which selectively antagonizes this same class of receptors. As shown in Fig. 6, ATPA (10 or 30 μM) did not modify 15 mM [K^+^]_e_-evoked [^3^H]GABA release from rat caudal brainstem synaptosomes; moreover, KA-induced potentiation of evoked release was not reversed by UBP310 (30 μM). UBP310 alone (30 μM) had no effect on spontaneous or [K^+^]_e_-evoked release of [^3^H]GABA.

**Figure 7:**
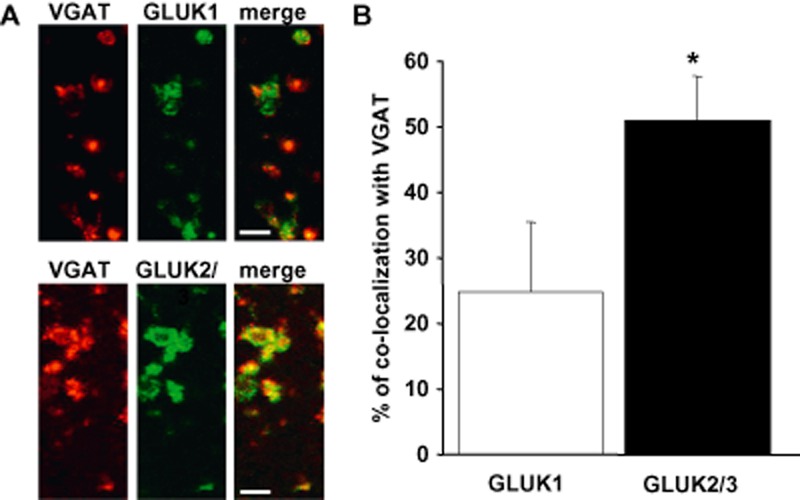
Differential expression of GLUK1 versus GLUK2/3 subunits in GABAergic synaptosomes prepared from the caudal brainstem region containing the trigeminal caudal nucleus as detected by confocal immunofluorescence. (A) Vesicular GABA transporter (VGAT) immunoreactivity is shown in red, whereas green was used for GLUKs staining. Scale bar: 5 μm. The data shown are representative of at least three different experiments. (B) Quantification of the data in panel A. For each experimental condition, about 1200 particles in at least eight microscopic fields were analysed. *p < 0.05.

To investigate the possible differential distribution of KA-receptor subunits in GABAergic nerve terminals within the TCN, we performed confocal double immunofluorescence experiments on synaptosomes prepared from the caudal brainstem containing the TCN. To this aim, synaptosomes were incubated with an antibody directed against the KA-receptor subunit GLUK1 or with an antibody able to recognize both KA-receptor subunits GLUK2 and GLUK3 (Lu et al., 2005). To identify GABAergic nerve terminals, synaptosomes were co-incubated with an antibody directed against the VGAT. As shown in Fig. 7, a larger proportion of VGAT-positive synaptosomal particles were labelled by anti-GLUK2/3 antibodies (49.10 ± 6.80%) when compared with those labelled by anti-GLUK1 antibodies (25.10 ± 10.60%). Moreover, 65.30 ± 10.70% of GLUK2/3-positive particles were GABAergic (VGAT positive), whereas 49.90 ± 12.10% of GLUK1-positive particles were also labelled by anti-VGAT antibodies. These data suggest that GABAergic nerve terminals in the TCN preferentially express KA receptors containing GLUK2/3 subunits.

## 4. Discussion

Transmission of nociceptive information requires a dynamic equilibrium between excitatory and inhibitory amino-acid neurotransmitter/receptor systems at both the spinal and supraspinal levels, and several forms of pathological pain have been attributed to reductions in the inhibitory tone (Gwak and Hulsebosch, 2011). In this study, we investigated the modulatory effects of excitatory neurotransmission on the release of GABA from nerve endings isolated from the TCN, the first central relay station for sensory information of orofacial origin (Hu et al., 1981).

In rat synaptosomes from the caudal brainstem area containing the TCN, we found that GLU potentiates the release of [^3^H]GABA evoked by 15 mM [K^+^]_e_, and this effect was reproduced by two naturally occurring GLU-receptor agonists, DO and KA, both of which displayed higher potencies when compared with GLU (DO>KA>GLU). None of the three GLU agonists modified the spontaneous release of [^3^H]GABA. The KA-induced enhancement of [^3^H]GABA release was completely abolished by NBQX, which antagonizes both AMPA and KA receptors (Sheardown et al., 1990), but it was not affected by the selective AMPA-receptor antagonist GYKI52466 (Wilding and Huettner, 1995). This result, together with the observation that other ionotropic GLU-receptor agonists such as AMPA or NMDA failed to exert GABA release-potentiating effects, suggests that KA receptors are involved in this response.

KA receptors are located at both the pre- and post-synaptic levels (Lerma et al., 2001; Huettner, 2003; Lerma, 2003). KA-receptor agonists have been shown to modify GABAergic and glutamatergic synaptic transmission in electrophysiological experiments and to regulate the release of neurotransmitters from synaptosomal preparations (for reviews, see Huettner, 2003; Lerma, 2003). These experiments involve bath applications of KA-receptor agonists, so it is sometimes unclear whether KA is exerting a direct effect on pre-synaptic KA receptors or whether GABA or GLU release is being regulated indirectly by some other mechanism. One of the problems that arise in interpreting the effects of KA-receptor agonists is that somatodendritic KA receptors are often present on pre-synaptic neurons. This situation can give rise to synaptic changes in different ways. For instance, KA stimulation of CA1 interneurons can trigger substantial GABA release that activates GABA_B_ receptors and depresses synaptic transmission (Frerking et al., 1999). There is also evidence that KA receptors in several CNS regions control cell excitability via a metabotropic mechanism that involves the activation of G-protein and intracellular signalling cascades (Rodríguez-Moreno and Lerma, 1998). Isolated, superfused nerve terminals are a valid experimental model for exploring the functional properties of the KA receptors that regulate GABA release. This approach eliminates problems related to effects mediated by dendritic or somatic receptors and the indirect effects that can be observed in cell cultures (Raiteri and Raiteri, 2000).

Thus far, five distinct KA-receptor subunits have been cloned, GLUK1-5. They co-assemble in various combinations to form functional receptors with distinct physiological and pharmacological properties (Jane et al., 2009). The results of our pharmacological experiments suggest that GLUK1 subunits are not involved in KA-induced potentiation of depolarization-evoked GABA release in the rat TCN. In fact, ATPA, which selectively activates KA receptors containing GLUK1 subunits (Brehm et al., 2003), had no effect on [K^+^]_e_-evoked release of [^3^H]GABA; in addition, KA-induced [^3^H]GABA release enhancement was not reversed by UBP310, a potent and selective antagonist of native and recombinant KA receptors that include GLUK1 (Dolman et al., 2005), further reinforcing this conclusion. It should be noted that UBP310 also exerts strong inhibitory effects on homomeric GLUK3 receptors and mild antagonism of GLUK2/GLUK3 heteromers (Perrais et al., [Bibr b34]); therefore, the fact that UBP310 was inactive in our experiments also suggests that the KA receptor whose activation results in GABA release potentiation is probably not a GLUK3 homomer, although it did not exclude the possibility that heteromeric GLUK2/GLUK3 receptors are involved. In addition, the fact that neither ATPA nor AMPA, both weak agonists of heteromeric GLUK2/GLK5 receptors (the most common KA-receptor subtype found in the brain) (Sakimura et al., 1992), failed to affect [^3^H]GABA release in our model tends to exclude a significant functional role for these receptors in the control of GABA release from TCN nerve terminals.

The plant lectin Con A is an allosteric activator which blocks desensitization of KA receptors consisting of GLUK1 or GLUK2 homomers (Huettner, 1990; Paternain et al., 1998), but it has no effect on agonist-induced desensitization of GLUK3 receptors (Schiffer et al., 1997). In TCN synaptosomes, Con A failed to modify the KA-induced potentiation of [^3^H]GABA release, arguing against the involvement of GLUK1 or GLUK2 homomeric pre-synaptic KA receptors; again, the possibility remains that GLUK2/GLUK3 heteromers might be implicated.

In our experiments, the agonist potencies of GLU and KA were both fairly limited. GLU activates homomeric GLUK1 and GLUK2 receptors with an EC_50_ in the low micromolar range (Alt et al., 2004), but its effects on GLUK3 receptors are much weaker (EC_50_ ∼5 mM) (Schiffer et al., 1997). KA and DO show little selectivity between GLUK1 and GLUK2 (Alt et al., 2004). KA binds to GLUK3 with high affinity (Ki value of 77 nM) (Bettler et al., 1992), but like GLU, it is a low-potency agonist of GLUK3 or GLUK3/GLUK5 receptors (Schiffer et al., 1997). DO is a potent GLUK1 and GLUK2 receptor agonist, and while it binds to GLUK3 receptors with high affinity (Sagot et al., 2008), it does not activate them (Schiffer et al., 1997). In our experiments, the low-potency agonism exhibited by GLU, DO and KA might point to the presence of GLUK3 subunits in the pre-synaptic KA receptors we examined. When this subunit is co-expressed with GLUK2, the responses to KA are markedly reduced (Cui and Mayer, 1999). Our pharmacological data suggest that pre-synaptic KA receptors expressed of GABAergic interneurons in the TCN do not contain GLUK1 and that they are more likely to be GLUK2/GLUK3 heteromers. This conclusion is consistent with the results of our immunofluorescence experiments showing that GABAergic nerve terminals in the TCN preferentially express GLUK2/GLUK3 rather than GLUK1 subunits. A similar relative distribution of KA-receptor subunits has been described in GABAergic nerve terminals of rat spinal dorsal horn (Lu et al., 2005), a region showing a structural organization similar to that of the TCN. Thus, morphological evidence corroborates the hypothesis of a preferential role of GLUK2/3 subunits in the pre-synaptic regulation of GABA release in the TCN.

According to our findings, the activation of a GLUK2/GLUK3 KA receptor facilitates [^3^H]GABA release from depolarized nerve terminals of the TCN, but it failed to affect basal [^3^H]GABA release. On the other hand, other authors (Nakamura et al., 2010) have shown that KA receptors containing the GLUK1 subunit can directly activate spontaneous GABA release from rat periaqueductal grey neurons, a phenomenon possibly explained by brain area-related differences in KA-receptor subtypes regulating GABA release. In addition, it should be emphasized that subunit composition influences the pharmacological and functional properties of the resulting KA receptor (Perrais et al., 2009). In fact, the deactivation phase of KA receptors is slower in GLUK1- than GLUK2-containing receptors (Swanson et al., 1997); thus, it seems likely that pre-synaptic GLUK2/GLUK3 receptors desensitize more rapidly than those that contain GLUK1. Thus, influx of Na^+^ ions through the GLUK2/GLUK3-containing receptors might depolarize the synaptosomes to values unable to trigger neurotransmitter release; therefore, the functional role of GLUK2/3-containing KA receptors would be only revealed when mild depolarizing stimuli are applied. This hypothesis seems consistent with the observed ability of pre-synaptic KA receptors to facilitate [^3^H]GABA release being inversely related to the magnitude of the depolarizing stimulus, since higher [K^+^]_e_ concentrations would activate voltage-gated calcium channels to a level sufficient to trigger substantial neurotransmitter release.

The involvement of KA receptors at many stages of synaptic transmission of noxious stimuli offers promising clinical perspectives. Several studies have provided evidence for the antinociceptive effects of compounds that antagonize GLUK1-containing KA receptors (Simmons et al., 1998; Dominguez et al., 2005), the key receptor expressed in neurons of dorsal root ganglia (Kerchner et al., 2002). Our findings indicate that the KA-receptor subtype modulating GABA release in the TCN is different from that expressed on glutamatergic terminals of primary afferent neurons, or from that described in the somatodendritic compartment of second-order neurons (Ruscheweyh and Sandkühler, 2002). Greater knowledge of the pharmacological properties of KA receptors capable of regulating inhibitory neurotransmission in the TCN could reveal novel pharmacological strategies to be explored for controlling migraine and/or facial pain.

## Author contributions

IS and VB performed experiments and analyzed the data. DC and PN provided critical revision of the article. MT contributed to data analysis and interpretation, as well as to the critical revision of the article. MM contributed to study conception, design and coordination, as well as to data analysis and interpretation, manuscript drafting, revision and final approval.
